# Heterogeneity of Hepatic Stellate Cells in Fibrogenesis of the Liver: Insights from Single-Cell Transcriptomic Analysis in Liver Injury

**DOI:** 10.3390/cells10082129

**Published:** 2021-08-19

**Authors:** Wenjun Zhang, Simon J. Conway, Ying Liu, Paige Snider, Hanying Chen, Hongyu Gao, Yunlong Liu, Kadir Isidan, Kevin J. Lopez, Gonzalo Campana, Ping Li, Burcin Ekser, Heather Francis, Weinian Shou, Chandrashekhar Kubal

**Affiliations:** 1 Division of Transplant Surgery, Indiana University, Indianapolis, IN 46202, USA; kisidan@iu.edu (K.I.); kevlopez@iu.edu (K.J.L.); glcampan@iu.edu (G.C.); pili@iupui.edu (P.L.); bekser@iu.edu (B.E.); 2 Department of Pediatrics, Indiana University School of Medicine, Indianapolis, IN 46202, USA; siconway@iu.edu (S.J.C.); liuying@iu.edu (Y.L.); psnider@iu.edu (P.S.); 3 Genome Editing Center, Indiana University School of Medicine, Indianapolis, IN 46202, USA; hanchen@iupui.edu; 4 The Center for Medical Genomics, Indiana University School of Medicine, Indianapolis, IN 46202, USA; Hongao@iu.edu (H.G.); yunliu@iu.edu (Y.L.); 5 Division of Gastroenterology, Department of Medicine, Indiana University School of Medicine, Indianapolis, IN 46202, USA; heafranc@iu.edu

**Keywords:** single-cell RNA sequencing, hepatic stellate cell sublineage, myofibroblast, liver fibrosis, carbon tetrachloride

## Abstract

Background & Aims: Liver fibrosis is a pathological healing process resulting from hepatic stellate cell (HSC) activation and the generation of myofibroblasts from activated HSCs. The precise underlying mechanisms of liver fibrogenesis are still largely vague due to lack of understanding the functional heterogeneity of activated HSCs during liver injury. Approach and Results: In this study, to define the mechanism of HSC activation, we performed the transcriptomic analysis at single-cell resolution (scRNA-seq) on HSCs in mice treated with carbon tetrachloride (CCl_4_). By employing *LRAT-Cre:Rosa26^mT/mG^* mice, we were able to isolate an activated GFP-positive HSC lineage derived cell population by fluorescence-activated cell sorter (FACS). A total of 8 HSC subpopulations were identified based on an unsupervised analysis. Each HSC cluster displayed a unique transcriptomic profile, despite all clusters expressing common mouse HSC marker genes. We demonstrated that one of the HSC subpopulations expressed high levels of mitosis regulatory genes, velocity, and monocle analysis indicated that these HSCs are at transitioning and proliferating phases at the beginning of HSCs activation and will eventually give rise to several other HSC subtypes. We also demonstrated cell clusters representing HSC-derived mature myofibroblast populations that express myofibroblasts hallmark genes with unique contractile properties. Most importantly, we found a novel HSC cluster that is likely to be critical in liver regeneration, immune reaction, and vascular remodeling, in which the unique profiles of genes such as Rgs5, Angptl6, and Meg3 are highly expressed. Lastly, we demonstrated that the heterogeneity of HSCs in the injured mouse livers is closely similar to that of cirrhotic human livers. Conclusions: Collectively, our scRNA-seq data provided insight into the landscape of activated HSC populations and the dynamic transitional pathway from HSC to myofibroblasts in response to liver injury.

## 1. Introduction

Liver cirrhosis is a major health burden in patients with liver diseases. It frequently occurs following chronic insults to trigger wound healing responses in the liver and induces the excessive deposition of extracellular matrix (ECM) [1]. The current understanding of liver fibrosis focuses on abnormal wound healing that occurs as a consequence of hepatic stellate cell (HSC) activation and the generation of myofibroblasts (activated HSCs or portal fibroblasts) [1]. At present, due to a lack of mechanistic clarity concerning the pivotal drivers of liver fibrosis, liver transplant remains the sole therapeutic option.

The HSC (also referred to as Ito cell, fat-storing cell, lipocyte, perisinusoidal cell) is one of the key nonparenchymal cell types of the liver. HSCs produce and secrete many different cytokines and growth factors that are critical for liver homeostasis in normal physiological conditions and control liver regeneration after an injury [2]. Upon liver injury, HSCs reduce the expression of genes such as peroxisome proliferator-activated receptor gamma (PPARγ) [3], lose lipid droplets, and activate into myofibroblasts by starting to express genes that encode contractile proteins and extracellular fibrogenic genes such alpha-smooth muscle actin (α-SMA) and collagen Type I, respectively. Upregulation of these genes is critical for wound healing in the reparative processes [1]. Persistent HSC to myofibroblast transformation and subsequent extracellular matrix (ECM) protein deposition is crucial for liver fibrosis. In addition to their proliferation and migration into the site of liver injury where they secrete ECM to maintain liver integrity, activated HSCs also play other essential roles such as in the release of different kinds of cytokines (Stellakines) to regulate inflammation, vasoactive response, angiogenesis, and promote migration of other hepatic lineage cells to the injury site for the reconstruction of the liver architecture and function [1]. Currently, whether these different roles are fulfilled by a single and homogeneous population of activated HSCs, or by different sublineage cells with diversified function remains elusive.

Single-cell RNA sequencing (scRNA-seq) has become one of the leading technologies to interrogate tissues and organs’ functional and structural complexity at a single-cell transcriptomic level. Recent application of scRNA-seq on normal and disease human and rodent liver has provided an unprecedented molecular detail on hepatic lineage cell diversities and revealed previously uncharacterized subtypes of liver endothelial cells, Kupffer cells, collagen-producing mesenchymal cells, hepatocytes and HSCs in disease/injured human and mouse liver [4,5,6,7,8,9]. Another recent study by Dobie et al. traced hepatic mesenchyme cells using *pdgfrb-BAC-eGFP* reporter mouse line, with the approach of scRNA-seq, they uncovered the spatial zonation of HSC cells across the hepatic lobule and demonstrated that central vein-associated HSCs as the primary collagen-producing cells in chronic CCl_4_ treatment injured mouse liver [10]. In this study, with scRNA-seq approach, we characterized *LRAT-Cre:Rosa26^mT/mG^* traced HSC-derived lineage cells from mouse livers following two weeks of exposure to carbon tetrachloride (CCl_4_). Our analysis further provides a detailed landscape of HSC transformation and uncovered previously unknown HSC subtypes and their functional heterogeneity, and further shed light on the dynamics of HSC-to-myofibroblasts transition in response to liver injury.

## 2. Experimental Procedures

### 2.1. Animals

All animal studies were performed following procedures approved by the Institutional Animal Care & Use Committee at Indiana University School of Medicine under the IACUC study # 18090MD/R/HZ. 8-week old *LRAT-Cre:Rosa26^mT/mG^* mice were used for HSCs isolation. Both *LRAT-Cre* [11] and *Rosa26^mT/mG^* [12] mouse lines have been described previously. 8-week-old male C57BL/6 mice were purchased from Harlan Sprague Dawley Inc (Indianapolis, IN, USA). Mice were housed in a temperature-controlled environment with a 12-h/12-h light/dark cycle. Mice weighing 20–25 g were gavaged with CCl_4_ (2 mL/kg) diluted in olive oil (1:1 ratio) or an equivalent volume of olive oil mixed with saline three times weekly for 2 to 4 weeks as described elsewhere [13]. The age and sex-matched control group mice were gavaged with the same volume of olive oil (~100 mL) as CCl_4_ treated mice.

### 2.2. HSC Cell Isolation and Purification

Mouse hepatic stellate lineage cells were isolated as previously described [14]. The isolated HSC lineage cells were further purified by Fluorescence-activated cell sorting (FACS) of GFP positive cells with FacsAria cell sorter (BD Biosciences).

### 2.3. Single-Cell Library Preparation and Sequencing

Chromium™ Single Cell 3′ Library & Gel Bead Kit v2, 4 rxns PN-120267, Chromium™ Single Cell A Chip Kit, 16 rxns PN-1000009, Chromium™ i7 Multiplex Kit, and 96 rxns PN-120262 kit were used for single cell cDNA library preparation. The Single-cell 3′ RNA-seq experiment is conducted using the Chromium single cell system (10× Genomics, Inc, Pleasanton, CA, USA) and the NextSeq500 sequencer (Illumina, Inc, San Diego, CA, USA). The single cell suspension was first counted on the Countess II FL for cell number, cell viability, and cell size. Depending on the quality of the initial cell suspension, the single cell preparation includes re-suspension, centrifugation, and filtration to remove cell debris, dead cells and cell aggregates. Appropriate number of cells were loaded on a multiple-channel micro-fluidics chip of the Chromium Single Cell Instrument (10× Genomics) with a targeted cell recovery of 5000. Single-cell gel beads in Emulsion containing barcoded oligonucleotides and reverse transcriptase reagents were generated with the v2 single cell reagent kit (10× Genomics). Following cell capture and cell lysis, cDNA was synthesized and amplified. Illumina sequencing library was then prepared with the amplified cDNA. The resulting library was sequenced on an Illumina NextSeq500 with paired-end reads 26 bp + 98 bp at the Medical Genomic Core of Indiana University School of Medicine.

### 2.4. Seurat Analysis and Linear Dimensional Reduction

The R package Seurat version 3.1.4 [15] was used for the analysis. This includes cell type/state discovery with graph-based clustering, cell cluster marker gene identification, and various visualization. QC metrics of library size, number of features/genes, and mitochondrial reads (based on median-absolute-deviation (MAD), 3 MAD used here) were calculated with scatter package [16]. This, together with the QC analysis in Seurat, was used to determine the parameters used for excluding low-quality cells. For data normalization, by default, Seurat applies a global-scaling normalization method, “LogNormalize.” It normalizes the gene expression measurements for each cell by the total expression and multiplies by a scaling factor (10,000 by default), and log-transform the results. Highly variable features (features with high cell-to-cell variation) were next identified, and unwanted sources of variation, such as technical noise or batch effect, were removed. All these were performed with the Seurat function “SCTransform”. PCA was performed on the above-scaled data. By default, only the variable genes identified by differential expression (DE) are used. The heatmaps were used to decide which principal components (PCs) to include for further downstream analyses.

### 2.5. Determine Statistically Significant Principal Components

Seurat clusters cells based on their PCA scores, with each PC essentially representing a ‘metagene’ that combines information across a correlated gene set. The number of PCs was determined before using it in the downstream analysis. The top 50 PC were obtained in the analysis and the heatmaps were also used to decide which PCs to include in the downstream analysis. Cell clusters were identified with the Seurat functions “FindNeighbors” and FindClusters” using a resolution of 0.6 and 24 PCs.

### 2.6. Human Liver Samples

Cirrhotic liver tissue was obtained from explant livers from patients undergoing liver transplants from 2018 to 2019 at the Division of Transplantation, Indiana University School of Medicine. The specimens were not linked to specific individuals by the investigators either directly or indirectly through coding systems. The specimens were not collected specifically for this study through an interaction or intervention with living individuals.

## 3. Results

### 3.1. Single-Cell Transcriptomic Analysis of LRAT-Cre Reporter Traced Hepatic Cells in CCl_4_-Treated Mouse

**Figure 1:**
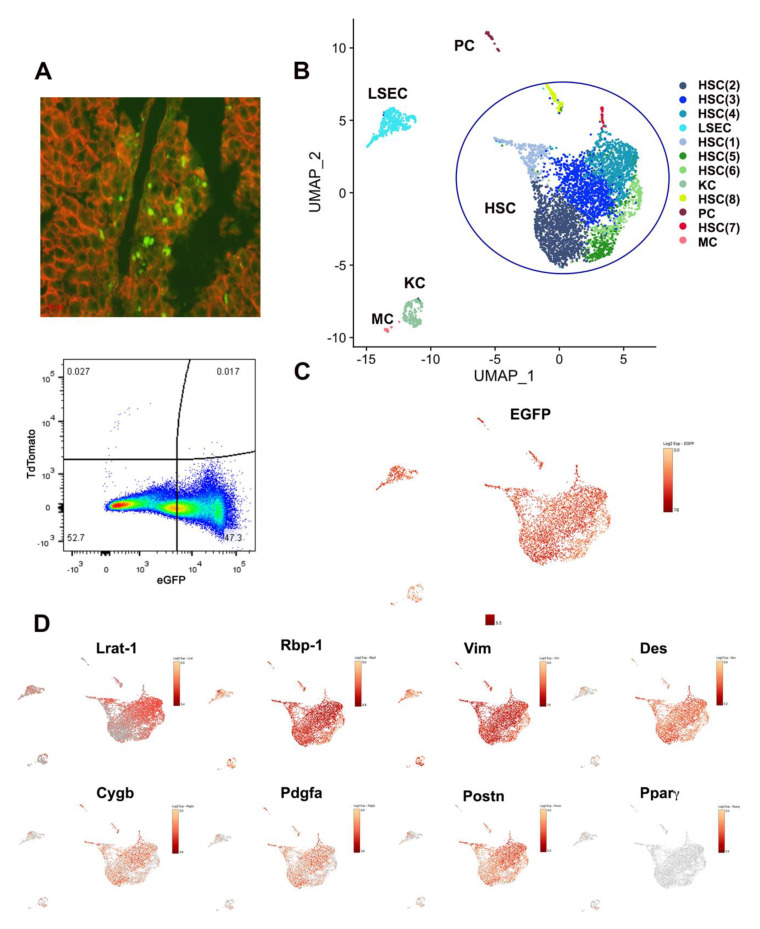
Single-cell RNA sequencing analysis of HSC population in CCl_4_ treated mouse liver. (**A**) upper panel, a representative image of mouse liver from CCl_4_-treated *LRAT-Cre:Rosa26^mT/mG^* dual-fluorescent reporter mouse, in which GFP positive cells shown in green fluorescence signals are *Lrat*-Cre traced cells in injured areas (100×); lower panel, a representative scatter FACS image of isolated GFP positive liver cells from CCl_4_-treated *LRAT-Cre:Rosa26^mT/mG^* mouse liver. (**B**) 2D Uniform Manifold Approximation and Projection (UMAP) visualization of single-cell clustering of filtered GFP positive cells from a CCl_4_-treated reporter mouse liver. Based on the signature gene expression profile, the color-coded identity of each cell cluster is defined on the right. HSC: hepatic stellate cells (HSC 1-8); KC: Kupffer cells; LSEC: liver sinusoidal endothelial cell; MC: mononuclear cells; PC: progenitor cell. (**C**) 2D UMAP visualization of GFP expression in all filtered cells within each cell cluster. (**D**) Representative Loupe images of HSC marker genes in all clusters.

**Figure 2:**
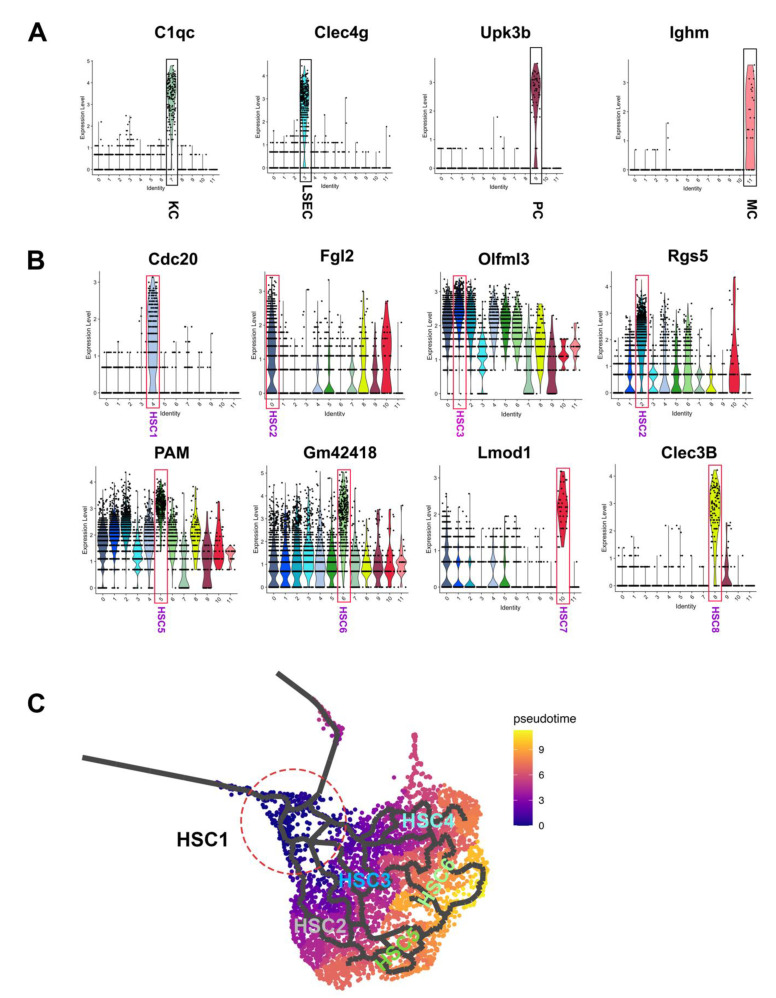
Heterogeneity of HSCs in CCl_4_ treated mouse liver. (**A**) Violin plot showing the expression pattern of marker genes to define non-HSC clusters, such as KC, LSEC, PC, and MC. (**B**) Violin plot showing the expression level of the representative marker genes in HSC clusters. (**C**) Monocle analysis overlayed all the HSCs onto UMAP, showing the transformation trajectories of single-cell HSC clusters from CCl_4_ treated mouse liver. HSC1 was used as root linage for analysis. Pseudo time is defined on the right.

To investigate the heterogeneity of activated HSCs during liver fibrogenesis, we performed scRNA-seq analysis on HSCs in mice treated with carbon tetrachloride (CCl_4_) for two weeks. This time point coincides with the early to middle stages of liver fibrosis compared to the common 4-week to 6-week CCl_4_ treatment that results in late-stage liver fibrosis and cirrhosis, which allowed us to capture a broader landscape of activated HSC population. The dual-color fluorescent, HSC-restricted, *LRAT-Cre:Rosa26^mT/mG^* reporter mice was employed to trace the HSC lineage derived cell population. Without Cre mediated recombination, cell membrane-localized tdTomato (mT) fluorescence is ubiquitously expressed in cells/tissues (Figure 1A, upper panel). However, in HSCs, Cre expression driven by Lecithin retinol acyltransferase (LRAT) promoter will lead to HSCs and their derived cell population to have *GFP* expression to replace red tdTomato (mT) fluorescence (Figure 1A, upper panel). High-purity and high-yield mouse HSC isolation procedure have been developed and reported previously [14], with the similar approach, we successfully isolate the activated HSCs from 2-week CCl_4_ treated mice and further enriched these cells by FACS sorting for GFP-positive cells (Figure 1A, lower panel). The single GFP positive cells were prepared and captured, and library preparation was carried out using the Chromium Single cell 3′ Reagent kits V2 (10× Genomics). Illumina sequencing libraries were prepared with the amplified cDNA. 26 bp of cell barcode and UMI sequences and 91 bp 3′-RNA reads were generated. A total of 5284 single-cell transcriptomes were obtained after removing low-quality cells with unique feature/gene counts over 6000 or less than 500, or >8% of the reads mapped to the mitochondrial genome (Figure S1A,B), and principal components analysis (PCA) (Figure S2A,B) was performed on the normalized and scaled data (Figure S2D,E). On average, 3482 genes were identified in each single cell (Figure S1A). Subsequently, the cells were projected onto a low-dimensional space encoding transcriptional state using UMAP to reduce the dimensionality for visualization. Heatmap analysis was also used to visualize the cell clusters. The results were categorized into 12 unique cell clusters from all filtered cells (Figure 1B and Figure S2F). By comparing to scCATCH [17] mouse liver database and using SingleR software package, the GFP positive cells were categorized into five major cell population: HSCs (4597 cells), liver sinusoidal endothelial cells (LSEC, 423 cells), Kupffer cell cluster (KC, 174 cells), progenitor cell cluster (PC, 61 cells) and monocyte (MC, 29 cells) (Figure 1B and Figure S2C). Furthermore, the signatures of differentially expressed genes attribute the HSC population into eight subtype clusters (designated as HSC1 to HSC8) (Figure 1B). When projecting GFP expression to UMAP, we found that GFP was present in all 12 clusters, which not only validated our isolation protocol but also confirmed that these non-HSC clusters were, in fact, *Lrat-cre* traced cells. Additionally, the feature plot demonstrated that some of the common HSC marker genes, such as retinol-binding protein 1 (Rbp-1) [18] and vimentin [2], were robustly expressed in all clusters. Interestingly, LRAT expression was dynamically distributed in HSC subtypes, robustly expressed in some HSC subclusters but relatively weak in others (Figure 1D). Several well-established HSC marker genes, including desmin (Des), Cygb, and Pdgfa showed stronger expression in HSC clusters (Figure 1D) compared to other cell clusters. Previously, it was reported that Gdf2 (also known as Bmp9), a member of the TGFb/BMP superfamily, was essential for mouse liver fibrogenesis induced by chronic CCl_4_ treatment [19]. Consistent with the observation, our analysis demonstrated that Gdf2 expression was found highly in most HSC clusters but was absent or expressed at a lower level in *LRAT-Cre* traced other liver cell clusters. In addition, LSEC cluster cells expressed common LSEC marker genes such as Clec4g (Figure 2A), Aqp1, GPihbp1, Fcgr2b, Ctla2a, Fam167b, Fabp4, and Egfl7 (Figure S3), KC cluster cells enriched for expression of KC maker gene and inflammatory response genes such as C1qc (Figure 1E), Lyz2, Lgals3, Wfdc17, Tyrobp and Ctss (Figure S4). PC cluster categorized by scCatch mouse liver database were positive for mesothelial markers, such as Upk3b ((Figure 2A), Krt19, Gpm6a, Mesothelin (Msln), Angptl7, Slpi, Lgfbp5, Lgfbp6, C3 (complement C3) and Crip1(Figure S6) [20,21], which suggested it as a mesothelial cell lineage cluster. Similarly, MC cluster cells categorized by scCatch mouse liver database express B cell marker gene such as CD79 and Ighm, which suggested it could be a B cell sublineage (Figure 2A and Figure S5).

### 3.2. Heterogeneity of Activated HSCs in CCl4 Injured Mouse Liver

**Figure 3:**
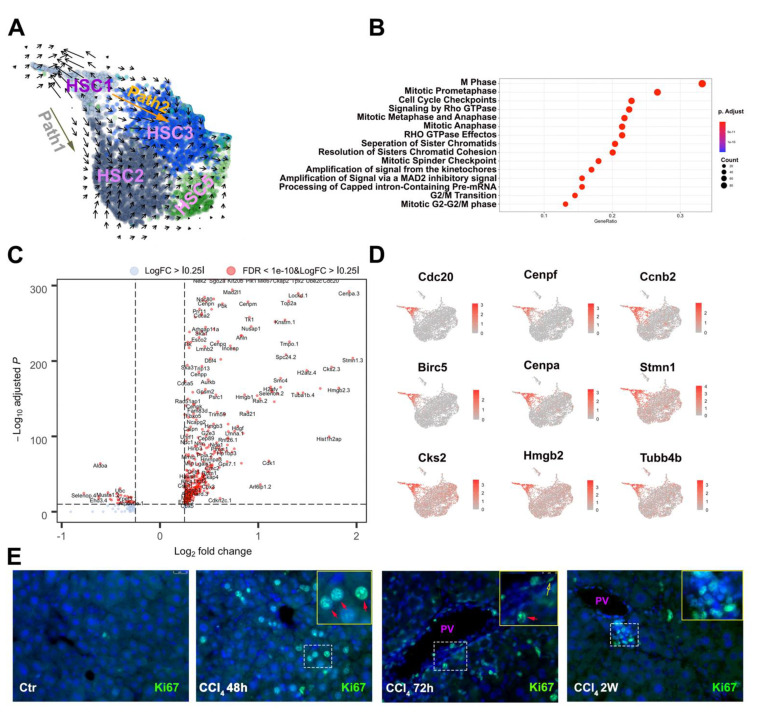
HSC1 as an early activated HSC subtype in response to CCl_4_ treatment. (**A**) scRNA velocity analysis overlaid on UMAP plot suggested the trajectory path from HSC1 to HSC2 and HSC3. (**B**,**C**) Reactome pathway analysis and volcano plot demonstrated, respectively, the activation of the pathways related to cytokinesis and the upregulation of the genes associated with cell proliferation. (**D**) Representative Loupe images of genes involved in cytokinesis in HSC1. (**E**) Representative images of Ki67 immunofluorescent staining of mouse livers isolated from CCl_4_-treat mice at indicated times (200×). Red arrows indicated the Ki67 staining positive hepatocytes; Yellow arrows indicated the Ki67 staining positive non-parenchymal cells.

Relative expression of selected cluster-specific genes demonstrated that Cdc20, Rgs5, Olfml3, Fgl2, Pam, Gm42418, Clec3b, and Lmod1 as the unique markers for different HSC clusters 1–8 (Figure 2B). We next sought to investigate the HSC transitioning trajectories in response to CCl_4_ exposure. Monocle analysis [22] was performed by projecting all the HSC sublineages cells onto UMAP to visualize the putative trajectories. As the HSC1 featured expression of genes that are associated with cell proliferation, such as Cdc20, suggesting it is at an early stage of HSC activation, we decided to set it as the root sublineage HSC. Our analysis revealed that, in pseudo time, HSC2, HSC3, and HSC4 as the main intermediated HSC clusters derived from HSC1, while HSC5 and HSC6 were the late to end-stage HSCs populations (Figure 2C). To confirm the above temporal trajectories prediction under pseudo time, we performed RNA velocity analysis, an algorithm based on the time derivative of the gene expression state [23], to predict potential directionality and speed of state transitions among all 12 hepatic cell clusters. As expected, velocity analysis showed no plain trajectory path among HSC, LSEC, KC, and MC. However, a potential trajectory path from PC to HSC clusters was suggested in the analysis (Figure S2G, note: the arrows in PC cluster pointing to HSC clusters). Consistent with the monocle analysis, velocity analysis also revealed that the HSC1 cluster may represent the earliest stage of the activated HSC population (Figure 3A). Four sets of major vectors, defined as paths, highlighted the HSC transition paths from HSC1 toward the other subtype HSC populations. Reactor pathway analysis demonstrated that the main activated pathways in the HSC1 transcriptome were associated with cytokinesis with high levels of expression in genes involved in M phase, mitotic pre-metaphase, cell cycle checkpoint, mitotic metaphase and anaphase, and mitotic anaphase. Volcano plot and feature plot further showed the specific upregulation mitotic related gene upregulation such as Cdc20, Ccnb2, Cenpf, Birc5, Cenpa, Stmn1, and Cks2 (Figure 3C,D). Among these genes, Cenpf encodes a protein that is a component of the nuclear matrix during the G2 phase of interphase [24], and Birc5 is a member of the inhibitor of apoptosis (IAP) gene family, which encode negative regulatory proteins that prevent apoptotic cell death [25]. Immunofluorescent staining of cell proliferative marker Ki67 confirmed HC is the major proliferating cell type at the acute stage of CCl_4_ insult, while the proliferation of HSC becomes prominent in mouse liver at the stage of 2-weeks of CCl_4_ treatment (Figure 3E). The identification of the proliferating HSC subtype in the CCl_4_ treated mouse liver provided the detailed transcriptomic insight of the HSC activation in the early stage of liver injury.

### 3.3. Two Major Differentiation Trajectory Paths Lead to Distinct Mature Myofibroblast Clusters

**Figure 4:**
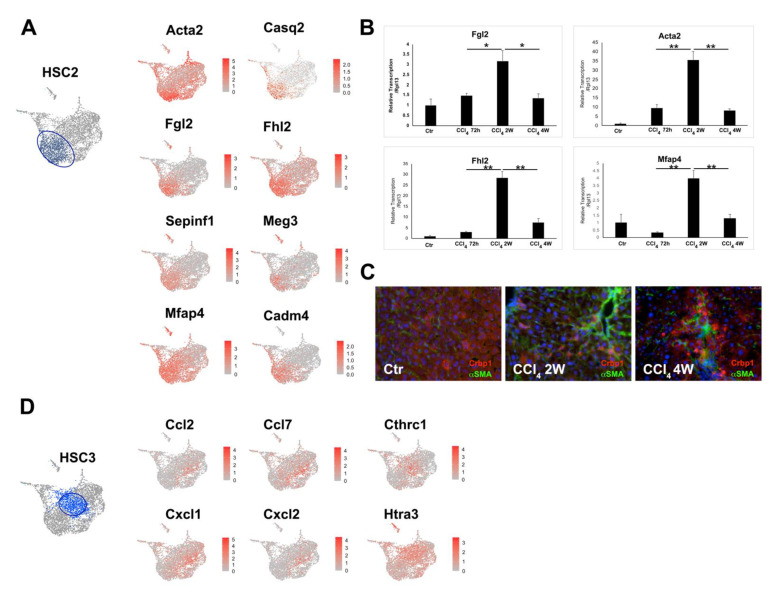
HSC2 and HSC3 are the two major HSC1 derived HSC subtypes in the CCl_4_-treated mouse liver. (**A**) Representative Loupe images of the expression pattern of the selected HSC2 marker genes. Each dot represents one cell. The blue dots (left) represent HSC2 cells. (**B**) qRT-PCR analysis of the representative HSC2 marker genes in mouse liver treated with CCl_4_ for 72-h, 2-week (2 w) and 4-week (4 w), respectively. *t*-test analysis, * *p* < 0.05, ** *p* < 0.01. (**C**) Co-Immunofluorescent staining of Crbp1 and aSMA2 in control (Ctr), 2 w and 4 w of CCl_4_ treated mouse liver (200×). (**D**) Representative Loupe images of the expression pattern of selected HSC3 marker genes. Blue dots (left) represent HSC3 cells.

Velocity analysis demonstrated two major trajectories, Path 1 and Path 2, leading to unique functional clusters. Path 1 appeared to represent the most common trajectory of HSC activation under CCl4 induced liver injury that results in a more mature HSC cluster, HSC2. A total of 1375 cells among all of the 3817 activated HSCs belonged to the HSC2 cluster, making it the largest HSC subtype. One of the top expression genes of the HSC2 cluster is Acta2, encoding a contractile protein aSMA and being regarded as the hallmark gene for myofibroblasts (Figure 4A). This cluster of cells expressed a high level of other genes that encode proteins relevant to myofibroblast contractile function, such as Tnnt2 and Casq2. Immunofluorescent staining confirmed that aSMA positive myofibroblasts were not co-localized with all of the Crbp-1 positive HSCs. Instead, these aSMA positive cells were more prominent in the fibrous area around the portal vein (Figure 4C) and in the bridging fibrosis area. Feature plot further revealed the top expressed genes of HSC2, such as Fgl2, Fhl2, Serpin f1, and Meg3 (Figure 4A). Reactome pathway analysis showed that the transcriptional alternation of the genes related to pathways such as cellular contraction, platelet activation, and collagen assembly et al., which suggested HSC2 is important for wound closure, cellular aggregation, and collagen organization in response to liver injury (Figure S7A). qRT-PCR analysis demonstrated that HSC2 marker genes, Acta2, Fgl2, Fhl2, and Mapf4 were robustly expressed in 2-week CCl_4_ treated mouse liver, but their expression dropped significantly in the mouse liver after 4-week CCl_4_ exposure (Figure 4B), which suggests that there is an attenuation of HSC2 population at the late stage of liver fibrosis.

Path 2 represented a trajectory of differentiation into inflammatory cluster HSC3. A total of 1146 activated HSCs belonged to the HSC3 cluster. This cluster featured specific expression of a group of chemokines, such as Cxcl1, Cxcl2, CCl2, and CCl7 (Figure 4D). These chemotactic molecules are well known for orchestrating inflammatory responses within the different organs [26]. Reactome pathway analysis further revealed that HSC3 is most likely the main HSC subtype for ECM organization, especially collagen biosynthesis and formation (Figure S7B). These findings indicated that HSC3 is a key HSC subtype that regulates inflammation and ECM deposition during liver remodeling process under injury conditions.

### 3.4. Unique HSC Differentiation Trajectory Path for Liver Repair and Vasoactive Modulation

**Figure 5:**
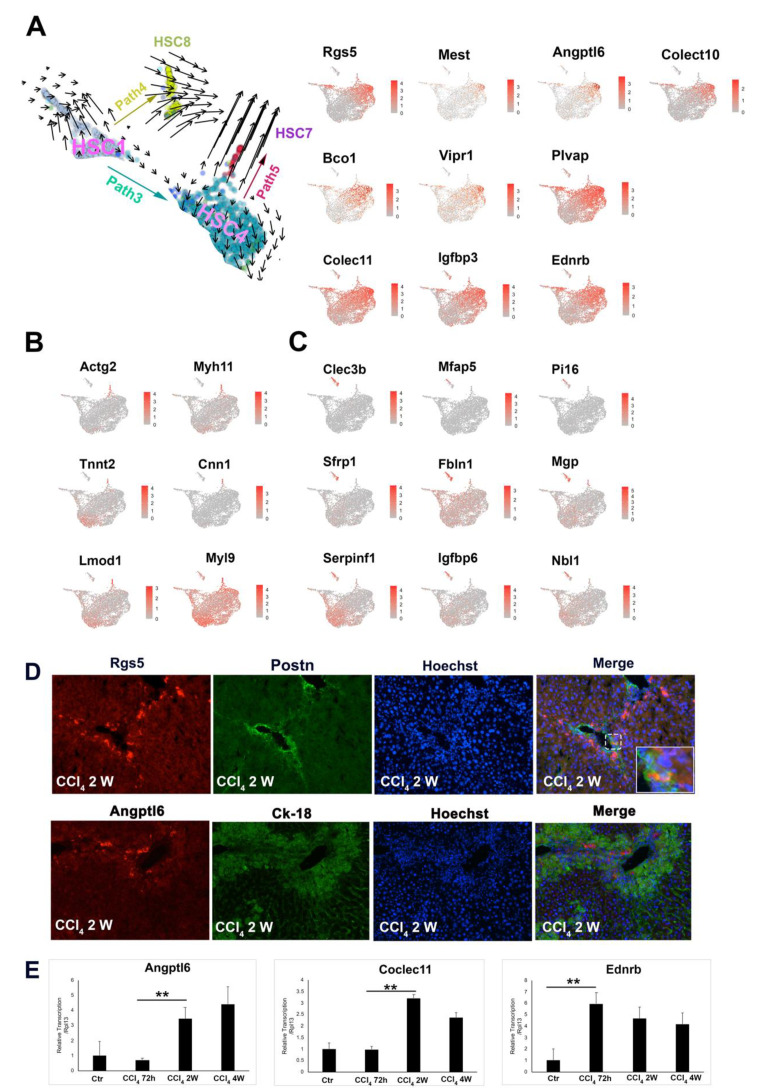
HSC4, HSC7, and HSC8 as novel HSC subtypes in CCl_4_-treated mouse liver. (**A**) Left panel, scRNA velocity analysis overlaid on UMAP plot suggested the trajectory path from HSC1 to HSC4 and HSC8, and HSC4 to HSC7 indicated by path3, path4, and path5, respectively; Right panel, Representative Loupe images of the expression pattern of representative HSC4 marker genes. (**B**,**C**) Representative Loupe images of the expression pattern of representative HSC7 and HSC8 marker genes, respectively. (**D**) Co-Immunofluorescent staining of Rgs5 and Postn (upper panels) and Angptl6 and Ck18 (lower panels) in CCl_4_-treated mouse liver (2-week) (100×). (**E**) qRT-PCR analysis of the representative HSC4 marker genes, Angptl6, Colec11, and Ednrb in the mouse liver treated with CCl_4_ for 72-h, 2-week (2 w), and 4-week (4 w). *t*-test analysis, ** *p* < 0.01.

Comparing to HSC2 and HSC3, Path3 represents differentiation trajectory of a new subtype of activated HSCs, HSC4, that featured with the expression of the genes such as Angptl6, Rgs5, Colec10, Colec11, Mest, Tmem56, Lrat, Ifitm1, Vipr1, Bco1, Plvap (C1), and Igfbp3 (Figure 5A). These genes have reported to be in association with tissue repair, vasoactive and angiogenesis. BMP10, another member of the TGFb/BMP family, may function as a homodimer or heterodimer with BMP9 to exert biological activities [27]. Interestingly, we found that unlike Bmp9 abundantly expressed across most of the HSC subtypes, Bmp10 was rather specifically expressed within the HSC4 cluster (Figure 5A). Reactome pathway analysis substantiated that HSC4 plays essential roles in governing the immune reaction during liver injury by regulating the interaction between lymphoid and non-lymphoid cells and the activation of the complement signaling cascade (Figure S7C). Co-immunofluorescence staining showed that Rgs5 positive HSCs were co-localized with a small portion of Postn positive HSCs in the portal vein and bridging fibrosis areas of 2-week CCl_4_ treated mouse liver section. In addition, Angptl6 positive HSCs were also identified at the portal vein (PV) region (Figure 5D). qRT-PCR analysis showed that Angptl6, Colect11, and Ednrb expression were persistent at the end of 4-week CCl_4_ exposure (Figure 5D), which suggested a potential role of HSC4 for mid and late-stage responses to liver injury.

### 3.5. Trajectory Paths That Contribute to the Obscure HSC Subtypes

In addition to the three major pathways that led to the differentiation of HSC1 into three major activated HSC clusters (HSC2–4), a small portion of HSC1 diverted into a different path that resulted in a small but unique population of HSCs, HSC8 (109 cells). This subtype of HSCs expressed a set of prominent fibroblast marker genes such as Clec3b, Mfap5, Pi16, Sfrp1(secreted Frizzled related protein1), Fbln1, Mgp, Gsn, Lgfbp6, Serpinf1, Nbl1, etc. (Figure 5C). Among them, Clec3b was detected in lung fibroblast cells and plays a role in tissue repair and wound healing [28]. Similarly, Pi16 was found to be mainly produced by fibroblasts and to regulate EC permeability and neuropathic pain [29]. This unique transcription signature suggested that this subtype of HSCs was essential for regulating neuropathic response and tissue repair. Interestingly, we found that some HSCs derived from HSC4 were further differentiated into the smallest population of HSCs, designated as HSC7 (39 cells). This subtype of HSCs selectively expressed high levels of genes related to cell contractility function, such as Actg2, Myh11, Myh9, Cnn1, and Tnnt2 (Figure 5B), and shared the same transcriptional profile genes as vascular smooth muscle cells, as reported by Dobie et al. [10]. Lastly, HSC5 and HSC6 are derived from HSC2 and HSC4, respectively. HSC6 sublineage cells expressed relatively higher levels of mitochondria genes such as mt-Co3, mt-Atp6, and mt-Cytb et al. (Figure S8), which indicated that this group of HSCs were either approaching to apoptosis/necrosis state or were in a special metabolic stress state [30]. Reactome pathway analysis indicated that the FGFR signaling, recycling pathway of L1, L1CAM interactions pathway were highly activated in HSC5 (Figure S9), and suggested HSC5 possesses a higher activity of cell axon growth during the wound healing process [31].

### 3.6. The Expression of CV HSC Markers and PV HSC Markers in HSC Subtypes

**Figure 6:**
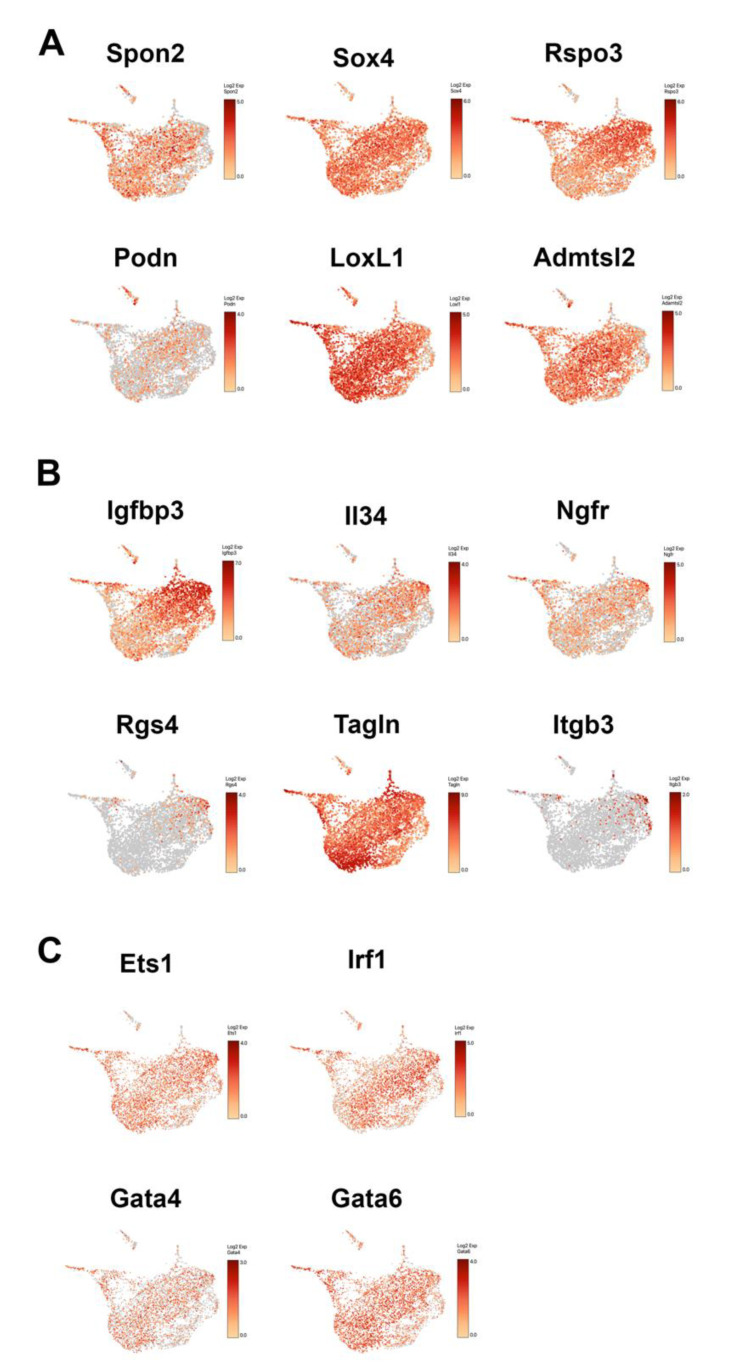
The comparison of the expression of CV HSC markers and PV HSC markers in HSC subtypes. (**A**) Representative UMAP Loupe images of the expression pattern of the selected CV HSC marker genes. Each dot represents a single cell. (**B**) Representative UMAP Loupe images of the expression pattern of the selected PV HSC marker genes. Each dot represents a single cell. (**C**) Representative UMAP Loupe images of the expression pattern of the selected HSC regulators, Ets1, Irf1, Gata4 and Gata6. Each dot represents a single cell.

Previously, with the approach of scRNA-seq using *pdgfrb-BAC-eGFP* as reporter mouse line, Dobie et al. uncovered HSC zonation across the healthy liver lobule, in which the portal vein-associated (PV) HSCs and the central vein-associated (CV) HSCs expressed a panel of distinguished marker genes [10]. When overlaying their findings with our scRNA-seq data, we found that the expression of PV HSC associated marker genes, such as Ngfr, Igfbp3, IL34 and Rgs4, are more closely associated with HSC3 and especially with HSC4 (Figure 6B), whereas the expression of CV HSC associated marker genes, such as Admtsl2, Spon2, Sox4 and LoxL1, covered a broader spectrum of HSC population such as HSC1, HSC2 and HSC3 (Figure 6A). These findings indicated that PV HSCs most likely gave rise to HSC4 and some of HSC3 cluster cells, and CV HSCs contributed to HSC1, HSC2 and HSC3, which are in line with the evidence from Dobie et al. [10] that CV HSC are the dominant collagen-producing cells in mouse model of centrilobular fibrotic liver injury. In addition, we compared the expression HSC lineage-determining regulators such as ETS1, IRF1 and GATA4/6 in all of the HSC subtypes (Figure 6C). Although these factors have been associated with quiescent (Ets1), inactivation (IRF1), activation (Gata4/6) of HSC [9], we found the expression of these regulators were distributed evenly among all HSC sublineages, which further suggested the expression of these genes in HSCs may be more spatial and temporal dynamic than we have appreciated in previous.

### 3.7. Heterogeneity of Activated Human HSC Population in Cirrhotic Liver

**Figure 7:**
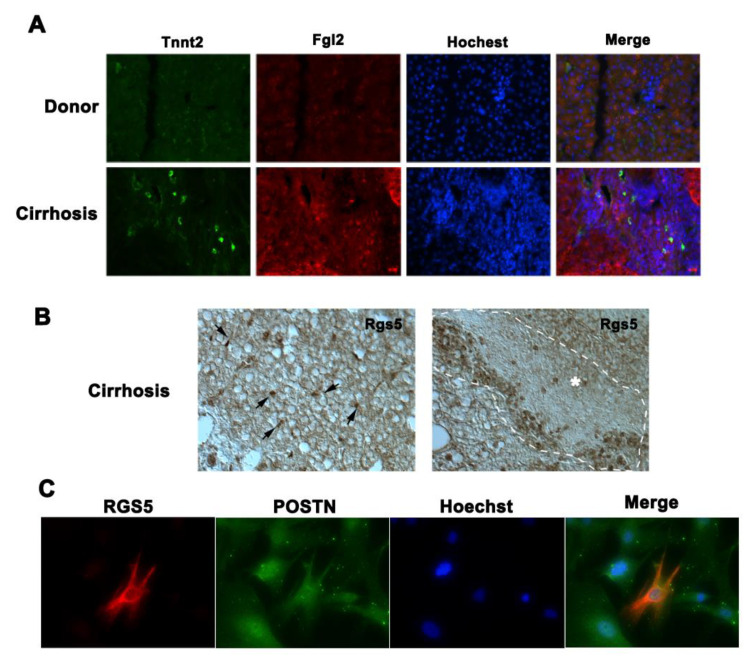
The analysis of heterogeneity of human HSCs in cirrhotic liver. (**A**) Co-Immunofluorescent staining of Tnnt2 and Fgl2 in control (Ctr) and cirrhotic human liver (200×). (**B**) Immunohistochemistry staining of Rgs5 in cirrhotic liver. Left panel shows interstitial Rgs5 positive HSCs (arrows) and right panel shows that Rgs5 positive signal detected in portion of activated HSCs in fibrotic area (within the dash line circle) (200×). (**C**) Co-Immunofluorescent staining of Rgs5 and Postn in fresh isolated human HSCs from cirrhotic liver (400×).

To further confirm whether a similar heterogeneity of HSCs is present in human liver fibrogenesis, we examined several murine-HSC cluster-specific markers in human cirrhotic livers. Fgl2 is a selective marker of HSC2 in CCl_4_-treated mouse livers, while Tnnt2 is the marker gene for the HSC7 sub-cluster and is also expressed at a lower level in a portion of HSC2 cells in the mouse liver. Immunofluorescent co-staining showed that while there was a large number of cells that expressed Fgl2, only a small number of cells were identified with robust Tnnt2 expression (hi-Tnnt2) in cirrhotic livers (Figure 7A). The hi-Tnnt2 cell populations were not co-localized with Fgl2 positive cells. Instead, Tnnt2 low expression cells were co-localized with Fgl2 signal. Moreover, immunohistochemistry staining revealed that Rgs5, a marker gene for activated HSC4 subtype in mouse liver, was detected in both sinusoidal HSCs and the bridging fibrosis area (Figure 7B) of human cirrhotic livers. POSTN is a common marker for activated HSCs [13]. Immunofluorescence co-staining demonstrated that the fresh isolated human HSCs from cirrhotic livers were positive for POSTN expression. However, only a small portion of POSTN positive cells also positive for RGS5 expression (Figure 7C). Collectively, these data strongly indicate that similar to mouse CCl_4_-injured model, there are heterogeneous HSC populations in the human cirrhotic liver. This finding further highlighted the complexity of human liver fibrosis.

## 4. Discussion

During liver injury, HSCs are the main cell type involved in liver fibrosis (1). Although purified HSCs produce collagen after activation in vitro, the precise contribution of HSCs to liver fibrosis and their function for liver tissue remodeling under injury conditions is not clearly defined in vivo. This is partly due to the lack of unique markers for HSCs as suggested previously, but also because the detailed functional transition mechanisms in HSC activation are not well understood. Conventional common knowledge is that liver injury induces HSCs activation and transforms them into myofibroblasts featuring the expression of a set of hallmark genes such as acta2 (encoding aSMA), Col1a1, CTGF, vimentin, etc. Myofibroblasts have been generally considered a homogeneous cell population that can be beneficial, such as in wound healing, and detrimental, such as in adverse tissue remodeling and contributing to a wide range of fibrotic liver diseases [32].

In this study, with the combination of genetic lineage tracing and scRNA-seq analysis, we analyzed the CCl_4_ treatment activated HSC lineage cells that traced with *LRAT-Cre:Rosa26^mT/mG^* reporter mouse and revealed the heterogeneous nature of activated HSCs with multiple subtypes that support a different aspect of wound healing processes including unfavorable liver tissue remodeling and fibrosis progression. Among these HSC sub-clusters, HSC1 is at an early phase of activation and featured with the expression of genes that relate to cytokinesis. This early activated HSC population is further differentiated into three major subtypes of HSCs as HSC2, HSC3, and HSC4. Among them, HSC4 is a subtype HSCs that was not fully recognized in previous studies. Among the selective top genes expressed in HSC4, Rgs5 encodes a member of the regulators of the G-protein signaling family that facilitates Gα_12/13_-mediated RhoA signaling, which is crucial for arteriogenesis and vasocontraction activity [33]. Recent publications demonstrated that Rgs5 expression was detected in skin and cardiac fibrosis under injury conditions [34,35], and RGS5 knock-in reporter mice specifically traced HSC in CCl_4_ injured liver [36]. Similarly, Mest is a negative regulator of wnt/beta-catenin signaling, and it was reported to be essential for muscle regeneration. Recent findings suggested that the reduction of Mest expression exacerbated CCl_4_ induced liver fibrosis [37]. Additionally, HSC4 cells selectively expressed the genes such as Ifitm1 [38] and Igfbp3 [39], implied that this population of cells had a critical role in innate immune responses. These observations clearly indicated that HSC4 is critical for mitigating the liver injury and fibrosis progression in liver disease. As a unique HSC subtype, HSC4 may also likely plays critical roles in angiogenesis, liver repair, inflammation, and vasoactive regulation by selectively expressing the genes such as *Angptl6, Colec10, Colec11, Mest, Tmem56, Lrat, Ifitm1, Vipr1, Bco1, Plvap*, and *Igfbp3*.

Acta2, one of the highly expressed genes in HSC2, has been a hallmark of myofibroblast [32] in various tissue injury settings, suggesting that HSC2 may represent mature myofibroblasts that are commonly acknowledged during livery injury. Consistently, HSC2 is the most populated HSC subtype among all the HSC sub-clusters we identified. This group also featured the expression of other genes related to myofibroblast contractile function, such as Tnnt2 and Casq2. In addition, several top markers of HSC2 implicated additional functions of this HSC subtype. Among them, Fgl2 was detected in activated HSC to regulate T-cell function in the patients with hepatic carcinoma, which also plays an important role in immune tolerance and immune response repression [40]. Fhl2 is another gene that highly selective expressed in HSC2 cluster cells. Previous findings indicated Fhl2 in attenuating lung inflammation during bleomycin-induced fibrosis [41]. Fhl2 deficiency was found to promote liver fibrogenesis [42]. Interestingly, while HSC2 expressed genes such as Fgl2 [40] and Fhl2 [41] that are critical for immune tolerance and inflammation repression, HSC3 featured by expressing of the chemokine genes such as Ccl2, Ccl7, Cxcl1, and Cxcl2, which suggests the different roles of HSC2 and HSC3 in inflammatory response under liver injury conditions. Additionally, the other top genes expressed in the HSC2 cluster, such as Serpinf1 (also known as PEDF) and Meg3, indicated that HSC2 cells may be critical for the regulation of angiogenesis during liver injury. Serpinf1 is a multifunctional secreted protein that previous findings suggested that it has anti-angiogenic, anti-tumorigenic, and neurotrophic functions [43]. Consistently, Meg3, a long non-coding RNA, was shown to be critical for regulation of angiogenesis, and Meg3-null led to an increased expression of angiogenic genes [44]. As HSC2 cells expressed anti-angiogenic genes such as Serpinf1 and Meg3 as their feature markers, while HSC4 subtype expresses Angptl6 and other genes for promoting angiogenesis, it may indicate that HSC4 and HSC2 regulate angiogenesis in the opposite direction during liver injury conditions to achieve vasculature homeostasis. These findings suggested a strong functional correlation, which is complimentary within the different subtypes of HSC, for wound healing and tissue repair in response to liver injury.

Lecithin-retinol acyltransferase (Lrat) is an enzyme that is critical for lipid drop formation [45]. Previously, the Tg mouse line, in which the Cre recombinase expression driven by *LRAT* promoter (*Lrat-Cre*) labeled the 99% of HSCs in the reporter mouse background under fluorescent microscopy analysis [11]. The same study also revealed that Lrat-Cre labeled vascular smooth muscle cells (VSMCs), which could be due to the fact that pericytes and VSMCs share a common precursor in the liver. Consistently, in this study, we identified HSC7 subcluster cells shared the similar transcriptional profile as VSMCs [10], which suggested the HSC derived cells contribute to VSMC lineage cells during liver injury. Consistent with these observations, Lrat protein was detected in both hepatic stellate cells and endothelial cells of normal rodents and human livers [46]. By tracing Lrat-Cre reporter mouse labeled hepatic lineage cells at a single-cell transcriptome level, we demonstrated that Lrat-Cre is capable of tagging the different HSC subtypes. However, around 12.7% (669 cells out of 5284 of filtered cells) of other hepatic cell types, including LSEC, KC, and PC (Mesothelial cells), were also labeled by their positive GFP expression activated by Lrat-Cre. This clearly suggested that there is a trace of Lrat promoter activity in the small portion of hepatic lineage cells other than HSCs. Previously, other HSC specific Cre transgenic lines, such as hGFAP-Cre mice, have also been reported to be able to label bile duct cells and cytokeratin 19 (CK-19) expressing cholangiocytes or bi-potent progenitor cells [2,47]. In addition to hepatic lineage cells, there were 29 cells (0.5%) out of the total of 5284 single-cell transcriptomes were identified as an independent cell cluster for expressing B cell marker genes such Cd79b and Ighm, as these cells are also positive for eGFP expression, we speculated that these cells could be captured by FACs as a result of non-specific Cre recombination.

The transformation of HSCs to myofibroblast during liver injury and liver diseases progression is a heavily studied area due to the profound clinical implications. However, the spatial and temporal cues for HSC activation and transformation are not well understood. Although our study is limited by a single time point analysis, which may not fully characterize the dynamic transformation of HSC during liver injury, it presents the insight of activated HSC populations in liver fibrogenesis. Given our study also demonstrated the heterogeneity of activated HSC in human cirrhotic livers, future studies at the single-cell transcriptome level at different stages of liver fibrosis may help the development of novel therapeutic strategies by targeting specific activated HSC subtype(s) to prevent or ameliorate liver fibrosis and reduce morbidity and mortality in various liver diseases.

## Data Availability

Data is contained within the article and supplementary material.

## Supplementary Materials

The following are available online at https://www.mdpi.com/article/10.3390/cells10082129/s1, Supplemental Experimental Procedures; Figure S1: QC and cell selection for further single-cell RNA seq analysis; Figure S2: Characterization of the common feature/genes of identified cell clusters; Figure S3: Violin plot showing the expression of the top marker genes of Lsec cluster relative to other cell clusters; Figure S4: Violin plot showing the expression of the top marker genes of Kupffer cell (KC) cluster relative to other cell clusters; Figure S5: Violin plot showing the expression of the top marker genes of monocyte (MC) cluster relative to other cell clusters; Figure S6: Violin plot showing the expression of the top marker genes of progenitor cell (PC) cluster relative to other cell clusters; Figure S7: Reactome pathway analysis of altered pathways in HSC2, HSC3 and HSC4 relative to other cell clusters respectively; Figure S8: Violin plot showing the expression of the top10 marker genes of HSC6 subtype relative to other cell clusters; Figure S9: Reactome pathway analysis of altered pathways in HSC5 subtype relative to other cell clusters.

## Abbreviations

HSC: hepatic stellate cell; Lrat: Lecithin-retinol acyltransferase; CCl_4_: carbon tetrachloride; LSEC: liver sinusoidal endothelial cells; KC: Kupffer Cell; MC: Monocyte; RGS5: Regulator of G protein signaling 5; Angptl6: Angiopoietin Like 6; Fgl2: Fibrinogen Like 2; Meg3: Maternally Expressed 3; Fhl2: Four and a Half LIM Domain 2; RBP-1: Retinol Binding Protein 1; CK-18: Cytokeratin 18; Ednrb: Endothelin Receptor Type B; Mfap4: Microfibril Associated Protein 4.
