# Supplementary material for: Heterogeneity of Hepatic Stellate Cells in Fibrogenesis of the Liver: Insights from Single-Cell Transcriptomic Analysis in Liver Injury
Source: Cells. 2021 Aug 19;10(8):2129. doi: 10.3390/cells10082129 (PMC8391930; doi:10.3390/cells10082129)
Supplement: Supplementary file 1 [file cells-10-02129-s001.zip › cells-1321887-supplementary.pdf]

Supplemental Information

Supplemental Experimental Procedures:

Mouse HSC isolation: Briefly, 2-week CCl_4 gavaged *LRAT-Cre:Rosa26$^{mT/mG}$* mice were anesthetized with inhalation of 1-3% (wt/vol) of isoflurane. Following laparotomy to expose inferior vena cava (IVC), the mouse liver was retrograde perfused in-situ with calcium-free Hank's Balanced Salt Solution (HBSS) containing 0.2mg/mL EDTA, followed by sequential perfusion with 0.4mg/mL pronase (Sigma, P5147) and 0.2% collagenase D (Roche, 11 088 882 001). After removal, the liver was minced and further digested with HBSS containing 0.2% collagenase D, 0.4 mg/mL pronase, and 0.1mg/mL DNase I (Roche, 10 104 159 001) in 37 ºC water bath for 20 min. Digestion was terminated with DMEM containing 5% serum. HSCs were further separated from hepatocyte and other hepatic cells with gradient centrifugation.

Analysis of sequence data: CellRanger 3.1.0 (http://support.10xgenomics.com) was utilized to process the raw sequence data generated. Briefly, CellRanger used bcl2fastq (https://support.illumina.com) to demultiplex raw base sequence calls generated from the sequencer into sample-specific FASTQ files. The FASTQ files were then aligned to the mouse reference genome mm10 with RNAseq aligner STAR. The aligned reads were traced back to individual cells, and the gene expression level of individual genes was quantified based on the number of UMIs (unique molecular indices) detected in each cell. The filtered feature-cell barcode matrices generated by CellRanger were used for further analysis.

Normalization, feature selection and data scaling: For data normalization, by default, Seurat applies a global-scaling normalization method "LogNormalize". It normalizes the gene expression measurements for each cell by the total expression and mutiplies by a scaling factor (10,000 by default), and log-transform the results. Highly variable features (features with high cell-to-cell variation) will be next identified. And unwanted sources of variation, such as technical noise, batch effect, will be removed. All these will be performed with the Seurat function "SCTransform".

Graph-based clustering and finding differentially expressed genes (cluster markers): "Seurat can find markers that define clusters via differential expression." Seurat continues to use T-distributed Stochastic Neighbor Embedding (t-SNE) and Uniform Manifold Approximation and Projection (UMAP) (1) for dimensional reduction and visualization to visualize and explore single-cell datasets. Seurat defines the cluster markers via differential expression. By default, it identifies positive and negative markers of a single cluster, compared to all other cells. The min.pct argument requires a gene to be detected at a minimum percentage in either of the two groups of cells, and the thresh.test argument requires a gene to be differentially expressed (on average) by some amount between the two groups. Setting both to 0 will dramatically increase in time of analysis since this will test a large number of genes that are unlikely to be highly discriminatory. Alternatively, max.cells.per.ident can be set to speed up the process. This will downsample each identity class to have no more cells than whatever this is set to. While there is generally going to be a loss in power, the speed increases can be significant and the most highly differentially expressed genes will likely still rise to the top". In the step below for maker genes identification, a gene must be expressed at least in 25% of the cells in one of the two populations compared and with a log fold difference greater than 0.25. The R package SingleR (2) and scCATCH (3) were used to annotate cell populations.

RNA velocity analysis and monocle trajectory analysis: RNA velocity analysis was performed using veloyto.py, velocyto.R v0.6 (4), and the R package SeuratWrappers. A loom file with the expression of spliced and unpliced mRNAs of each gene in each cell was generated with the BAM file from the CellRanger analysis. The loom file was converted to a seurat object and the spliced and unspliced data were added as assays to the seurat object from the above analysis. RNA velocity was estimated using the gene-relative model with k=25 cell kNN pooling and 2% fit.quantile for gamma fit. The resulting velocity estimates were projected onto the UMAP embedding from the Seurat analysis. To infer developmental trajectories, Seurat and Monocle 3 (5) were utilized. The Seurat object was converted to

SingleCellExpression object using the function as.SingleCellExperiment in Seurat. The SingleCellExpression object was then used to generate Monocle3 CDS object. The gene-cell counts, dimensional reductions and clusters determined from the Seurat analysis were directly imported into the Monocle CDS object. Trajectory graph was learned from the CDS object and the cells were ordered in pseudotime along the learned trajectory.

Immunofluorescence (IF) and Immunochemistry (IHC) Staining: For immunofluorescent staining, mouse or human liver tissues were fixed, embedded, and sectioned to 7μm thickness using standard techniques. Frozen sections were permeabilized with 0.1% Triton X-100 in PBS and then blocked in 5% normal goat serum, followed by incubation in primary antibody solution overnight at 4ºC, and subsequently in secondary antibody solution at room temperature for one hour. Sections were mounted in VECTASHIELD Antifade Mounting Medium (Vector Laboratories, H-1000). Immunofluorescent staining Images were taken with a Leica DMi8 microscope and Leica DFC7000 T imaging system. In the case of IHC staining, paraffin-embedded liver sections were then blocked with 1:200 goat serum 5% BSA in PBS for 1 hr at RT after blocking endogenous peroxidase using 3% H_2O_2 (in methanol) for 30 min. Then, sections were incubated with indicated primary antibodies overnight at 4 ºC, after which Vectastain ABC kit (Vector Laboratories, Inc) was used, following the manufacturer's instructions. Sections were incubated with 33-diaminobenzidinetetracloride (DAB) and counterstained with hematoxylin. The following antibodies were used in IF and IHC staining: anti-aSMA (Sigma, A-2547, 1:400), Anti-RBP1(ThermoFisher, PA5-28713, 1:100), anti-Angptl6 (ThermoFisher, PA5-31083, 1:100), anti-CK18 (Abcam, ab668, 1:100), anti-Cardiac Troponin T (ThermoFisher, MA5-12960, 1:200), Anti-Rgs5 (Sigma, HPA001821, 1:100), anti-Ki67(abcam, ab15580, 1:100), and anti-Postn (R&D system, MAB-3548, 1:100).

qRT-PCR analysis: Total RNA extraction and isolation from control or CCl_4 stimulated mouse liver were performed using Trizol™ reagent and a PureLink RNA kit (Invitrogen, Waltham, MA). cDNA were

synthesized with a SuperScript™ First Strand Synthesis System (Invitrogen). Quantitative PCR was performed using SsoAdvanced Universal SYBR Green Supermix (Bio-Rad, Hercules, CA). The relative expression level was normalized to the housekeeping gene HPRT1 or GAPDH. The mRNA expression levels of each gene were calculated with the $2^{-\Delta\Delta CT}$ method. The following primers were used in qRT-PCR analysis: Acta2: forward, 5′-GCTGAAGTATCCGATAGAACACG-3′, backward, 5′-GGTCTCAAACATAATCTGGGTCA-3′; Angptl6: forward, 5′- GGAATTGCCGCAAACCTCAC-3′, backward, 5′-CCTTCTGCGGGGACAAAACT-3′; Colec11: forward, 5′-TGGACAACCAGGTCACTCAA-3′, backward, 5′-AGCCTGTGCCAGGTATGAAG-3′; Ednrb: forward, 5′-CAATCCTCTGTATTTGGTGAG-3′, backward, 5′-CGTGATCGTTGGCTTTGAAC-3′; Fgl2: forward, 5′-TAGCCTGTTAGACCGCGATAC-3′, backward, 5′- CCTTACCATGCCTTTCCCCAA-3′; Fhl2: forward, 5′-GTCCTACAAGGATCGGCACT-3′; backward, 5′- ACAGGTGAAGCAGGTCTCGT-3′; Mfap4: forward, 5′- CTCCTCTACAAAGTGGCAGC-3′, backward, 5′- CTTTTCTGGAAAACCGTCCACT-3′.

Statistical Analysis: All values are presented as mean±standard error of the mean. Statistical significance was determined by *t* test (for groups of 2), by using Sigma plot software package (Sigma).

Supplemental Figures:

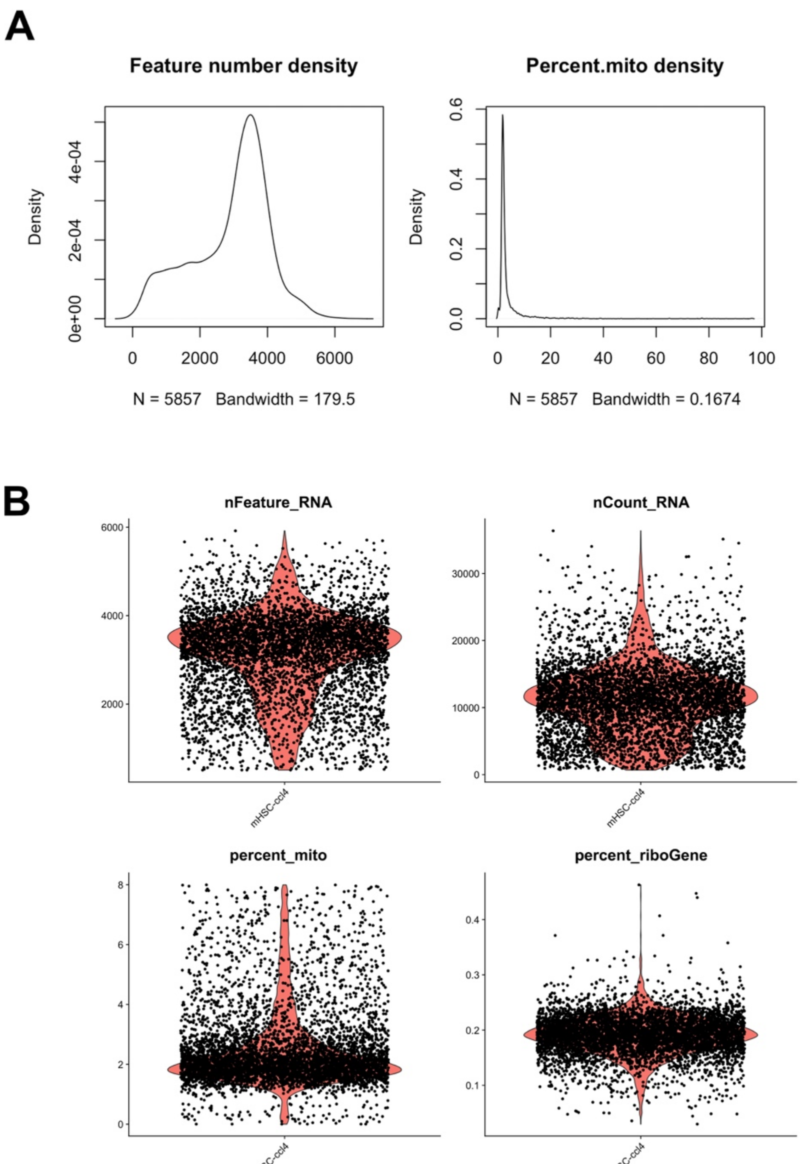

Figure S1. QC and cell selection for further single-cell RNA seq analysis. **A**. Density plot showing the density of feature/gene number and mitochondrial gene percentage in all sequenced cells. **B**. Violin plot displayed the number of feature/genes, UMIs, percentage of mitochondrial genes, and content ribosome protein-coding genes in all cells. Cells with unique feature/gene counts over 6000 or less than 500, or > 8% reads mapped to mitochondrial genome were excluded for further analysis.

A B

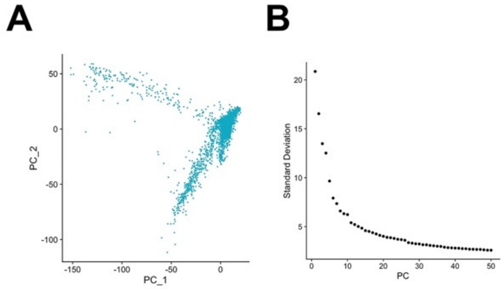

D E

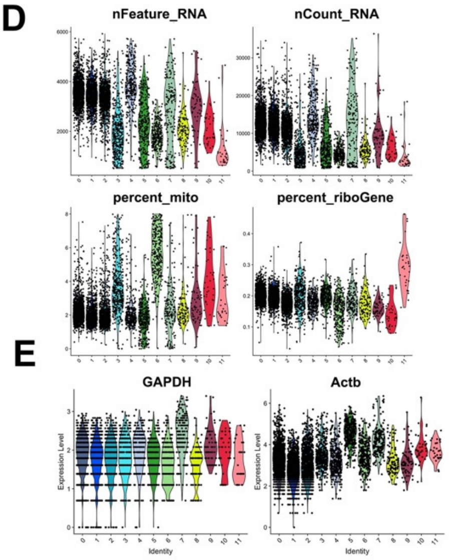

C

mHSC-ccl4			
Number of cells in each cluster/cell type			
cluster	Cluster annotation	Number of cells	percentageCellNumber
0	HSC (2)	1375	26.02
1	HSC (3)	1146	21.69
2	HSC (4)	974	18.43
3	LSEC	423	8.01
4	HSC (1)	331	6.26
5	HSC (5)	321	6.07
6	HSC (6)	302	5.72
7	KC	174	3.29
8	HSC (8)	109	2.06
9	PC	61	1.15
10	HSC (7)	39	0.74
11	MC	29	0.55

F G

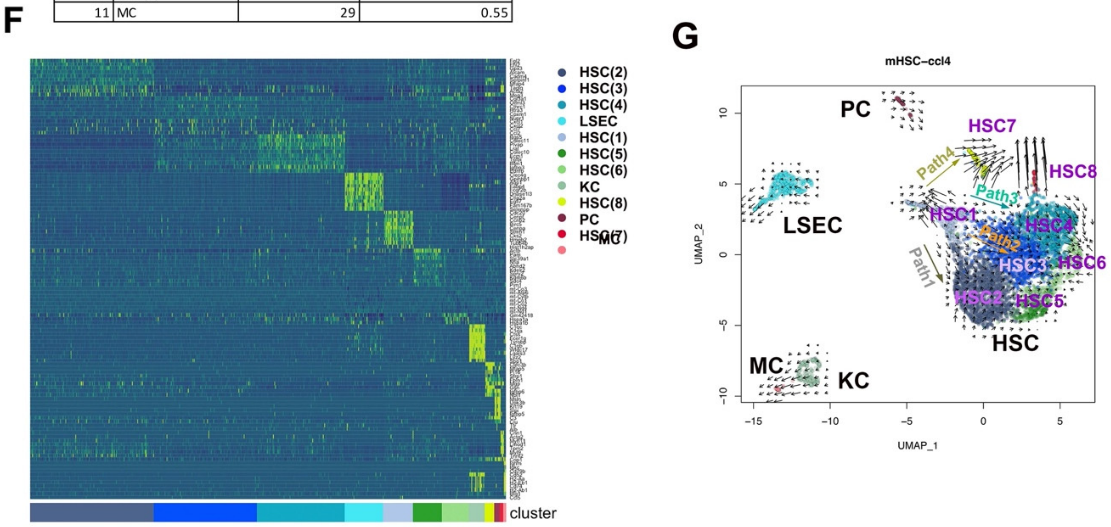

Figure S2. A and **B** Linear dimension reduction and Elbow plot analysis to determine the significant principal components of all the filtered cells. **C.** Cell number and percentage of all the identified cell clusters. **D**. Violin plot displays the expression distribution of the number of features (nFeature), the number of count (nCount), the percentage of mitochondria genes (percent.mito), and the percentage of ribosome genes across all the clusters. **E.** Violin plot displays the expression distribution of the housekeeping genes Acta and Gapdh across all the clusters. **F**. Heatmap showing the top 10 marker genes in each cluster of cells. **G**. scRNA velocity analysis overlaid on UMAP plot. Direction indicates the

transition toward the computed future state of a cell subtype. The sets of vectors, defined as paths, were illustrated by an arrow to indicate the HSC transition paths from HSC1 toward the other subtype HSC populations.

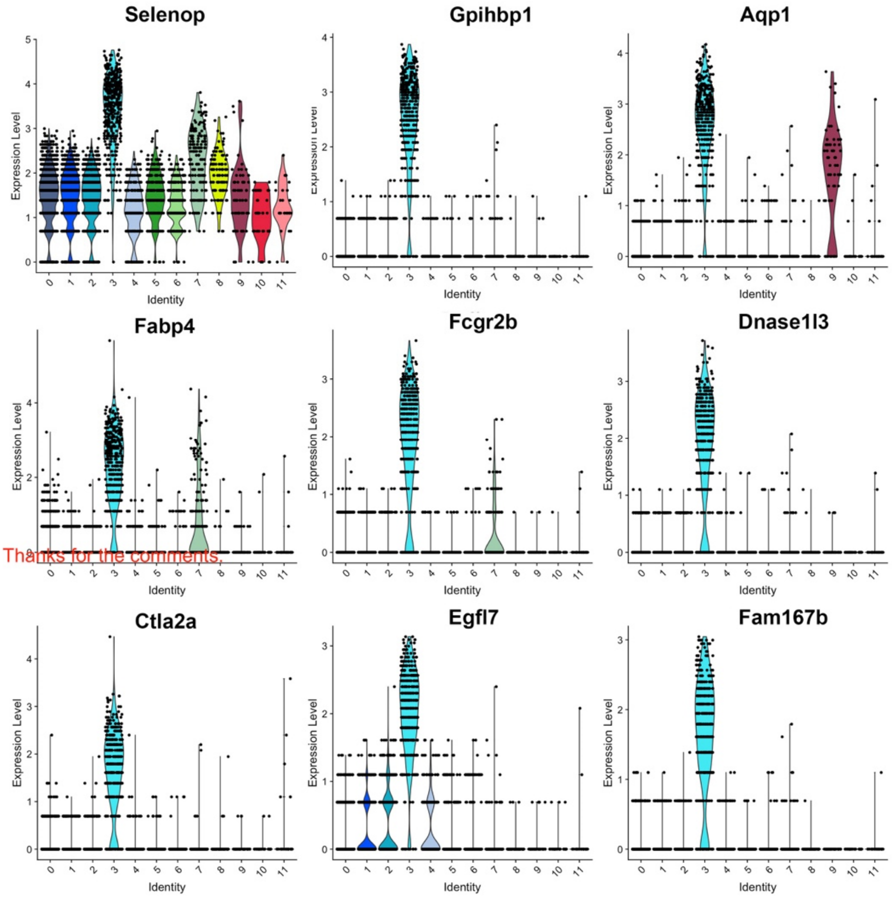

Figure S3. Violin plot showing the expression of the top10 marker genes of Lsec cluster relative to other cell clusters.

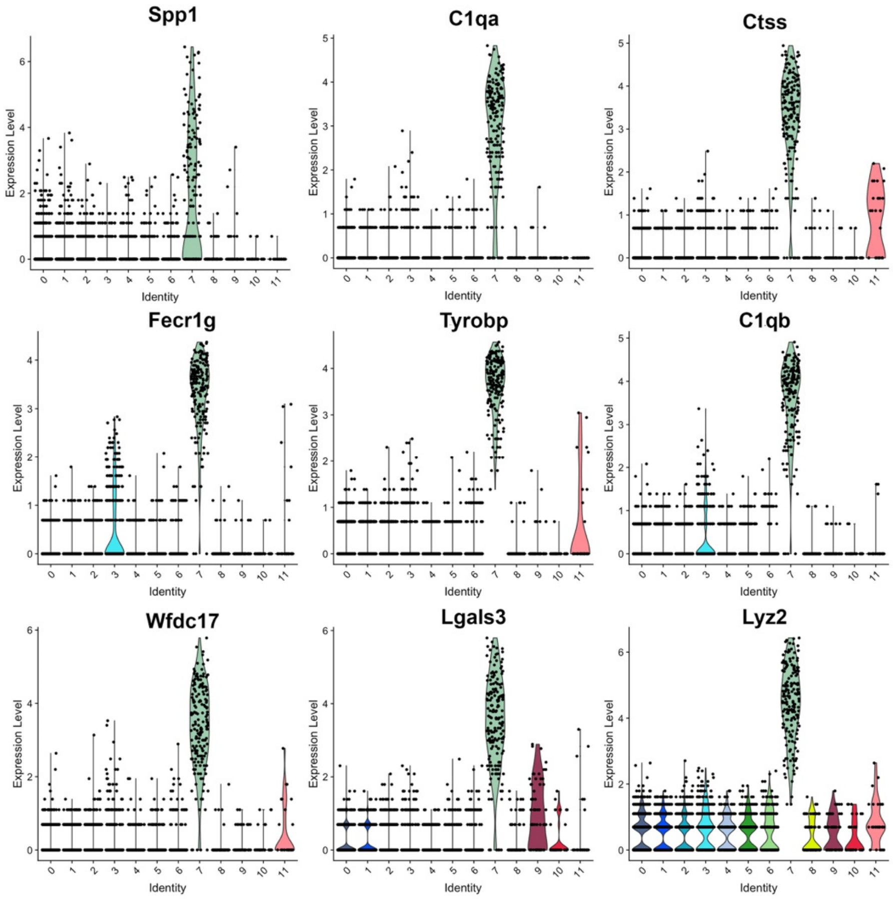

Figure S4. Violin plot showing the expression of the top10 marker genes of Kupffer cell (KC) cluster relative to other cell clusters.

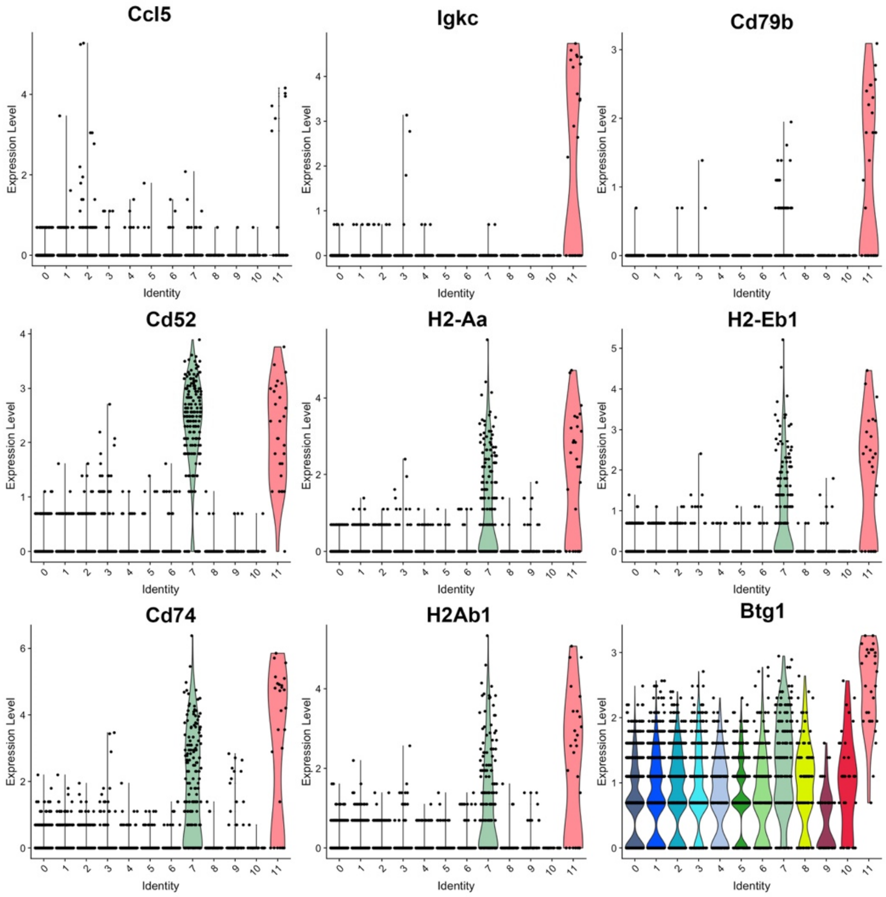

Figure S5. Violin plot showing the expression of the top10 marker genes of monocyte (MC) cluster relative to other cell clusters.

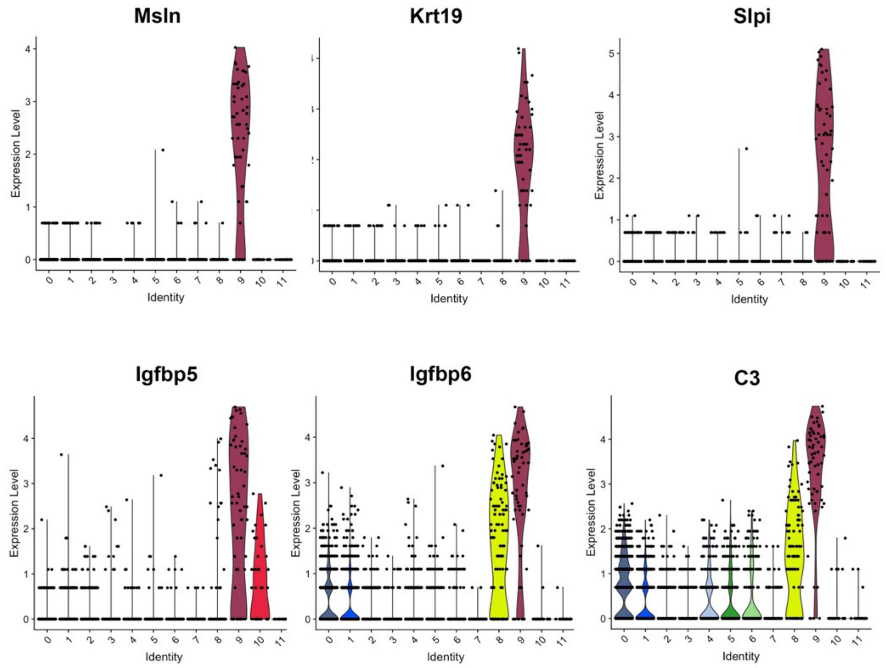

Figure S6. Violin plot showing the expression of the top10 marker genes of progenitor cell (PC) cluster relative to other cell clusters.

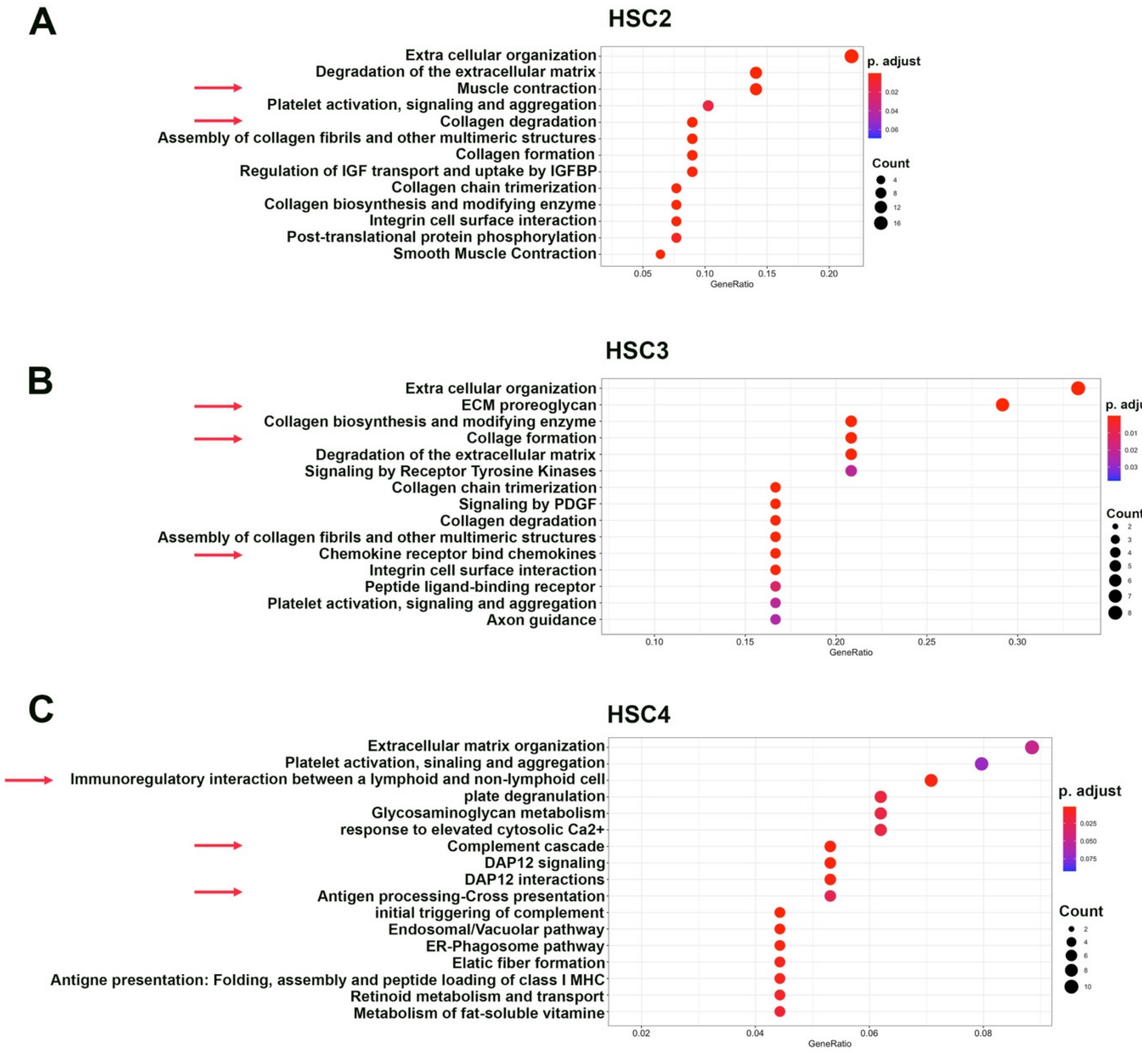

Figure S7. Reactome pathway analysis of altered pathways in HSC2, HSC3 and HSC4 relative to other cell clusters respectively. Alternated signaling pathway in HSC2 (**A**), HSC3 (**B**) and HSC4 (**C**) related to muscle contraction, collagen degradation, ECM proteoglycans, collagen formation, chemokine receptor bind chemokines, immunoregulatory interaction between a lymphoid and non-lymphoid cell, complement cascade and antigen processing-Cross presentation are indicated by arrow respectively.

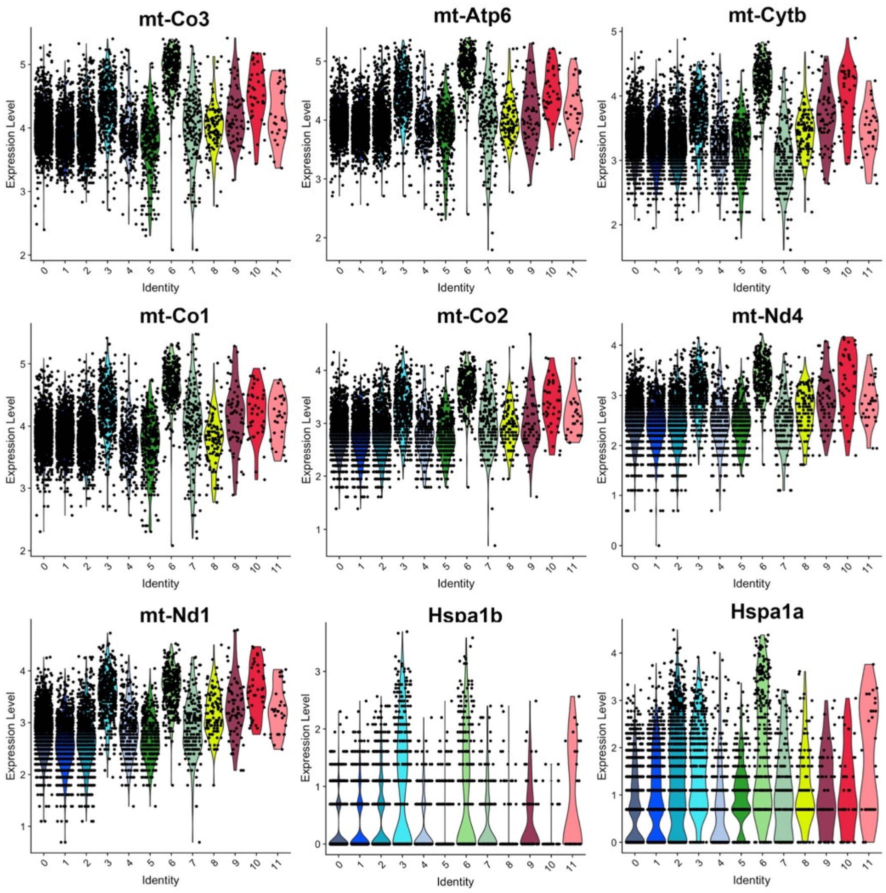

Figure S8. Violin plot showing the expression of the top10 marker genes of HSC6 subtype relative to other cell clusters.

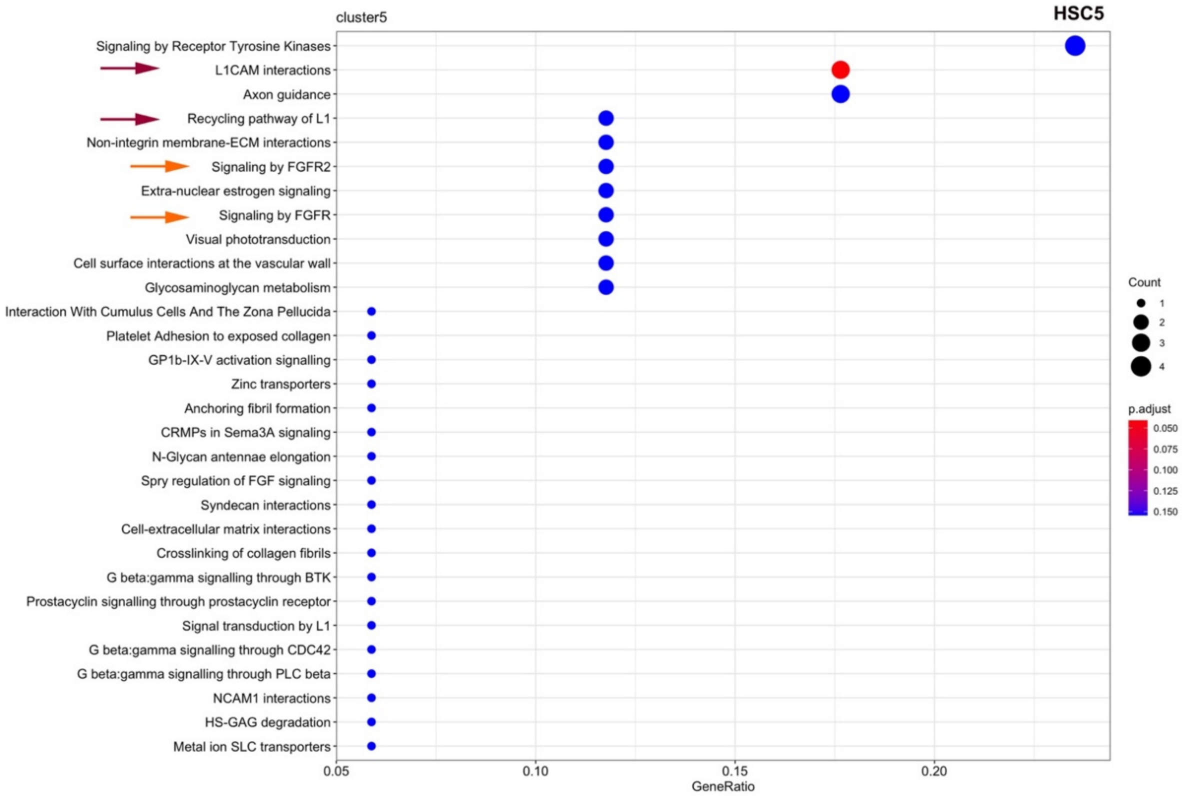

Figure S9. Reactome pathway analysis of altered pathways in HSC5 subtype relative to other cell clusters. L1CAM interaction and Recycling pathway of L1 are indicated by red arrows and signaling by FGFR2 and FGFR are indicated by arrows in orange.

References

1. Becht E, McInnes L, Healy J, Dutertre CA, Kwok IWH, Ng LG, Ginhoux F, et al. Dimensionality reduction for visualizing single-cell data using UMAP. Nat Biotechnol 2018.
2. Aran D, Looney AP, Liu L, Wu E, Fong V, Hsu A, Chak S, et al. Reference-based analysis of lung single-cell sequencing reveals a transitional profibrotic macrophage. Nat Immunol 2019;20:163-172.
3. Shao X, Liao J, Lu X, Xue R, Ai N, Fan X. scCATCH: Automatic Annotation on Cell Types of Clusters from Single-Cell RNA Sequencing Data. iScience 2020;23:100882.
4. La Manno G, Soldatov R, Zeisel A, Braun E, Hochgerner H, Petukhov V, Lidschreiber K, et al. RNA velocity of single cells. Nature 2018;560:494-498.
5. Cao J, Spielmann M, Qiu X, Huang X, Ibrahim DM, Hill AJ, Zhang F, et al. The single-cell transcriptional landscape of mammalian organogenesis. Nature 2019;566:496-502.
